# Crystal structure of *N*′-hy­droxy­pyrimidine-2-carboximidamide

**DOI:** 10.1107/S1600536814020285

**Published:** 2014-09-13

**Authors:** Nithianantham Jeeva Jasmine, Packianathan Thomas Muthiah, Nithianantham Stanley

**Affiliations:** a School of Chemistry, Bharathidasan University, Tiruchirappalli 620 024, Tamil Nadu, India

**Keywords:** crystal structure, pyrimidine-2-carboximidamide, non-covalent inter­actions, hydrogen bonding, π–π stacking inter­actions, biological activity

## Abstract

The title compound, C_5_H_6_N_4_O, is approximately planar, with an angle of 11.04 (15)° between the planes of the pyrimidine ring and the non-H atoms of the carboximidamide unit. The mol­ecule adopts an *E* configuration about the C=N double bond. In the crystal, adjacent mol­ecules are linked by pairs of N—H⋯O hydrogen bonds, forming inversion dimers with an *R*_2_^2^(10) ring motif. The dimers are further linked *via* N—H⋯N and O—H⋯N hydrogen bonds into a sheet structure parallel to the *ac* plane. The crystal structure also features N—H⋯O and weak C—H⋯O hydrogen bonds and offset π–π stacking inter­actions between adjacent pyrimidine rings [centroid–centroid distance = 3.622 (1) Å].

## Related literature   

For details of non-covalent inter­actions, see: Desiraju (2007 ▶). For the role of inter­molecular hydrogen bonds in the design of organic crystals, see: Aakeroy & Seddon (1993 ▶). For background to substituted *N*′-hy­droxy­benzamidines as inter­mediates in the synthesis of 1,2,4-oxa­diazole derivatives, see: Kundu *et al.* (2012 ▶). For the biological activity of substituted *N*′-hy­droxy­benzamidines and 1,2,4-oxa­diazole derivatives, see: Sakamoto *et al.* (2007 ▶); Tyrkov & Sukhenko (2004 ▶).
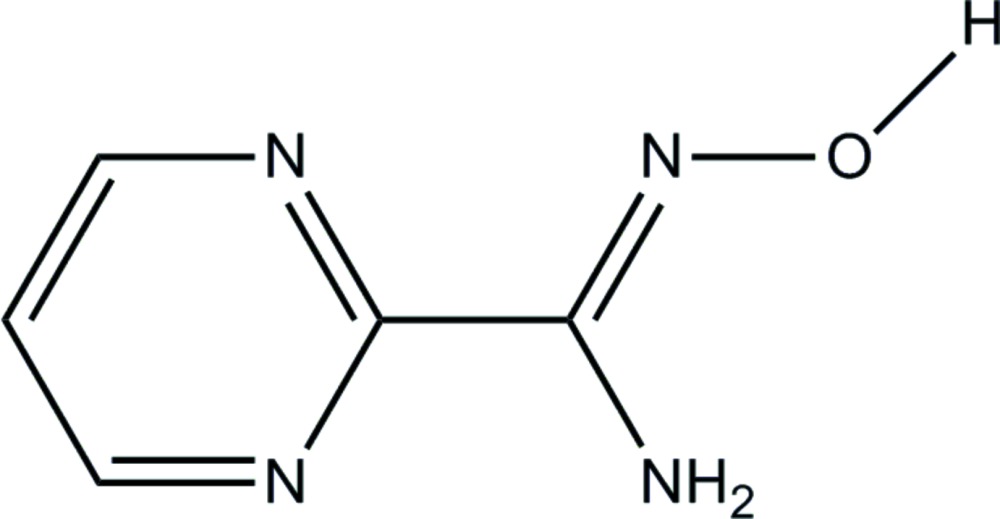


## Experimental   

### Crystal data  

C_5_H_6_N_4_O
*M* *_r_* = 138.14
Monoclinic, 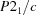

*a* = 7.4066 (7) Å
*b* = 8.0165 (8) Å
*c* = 10.2200 (9) Å
β = 101.888 (6)°
*V* = 593.8 (1) Å^3^
*Z* = 4
Mo *K*α radiation
μ = 0.12 mm^−1^
*T* = 100 K
0.62 × 0.17 × 0.08 mm

### Data collection  

Bruker SMART APEXII CCD area-detector diffractometer
Absorption correction: multi-scan (*SADABS*; Bruker, 2009 ▶) *T*_min_ = 0.931, *T*_max_ = 0.990
4073 measured reflections
1030 independent reflections
831 reflections with *I* > 2σ(*I*)
*R*_int_ = 0.047

### Refinement  

*R*[*F* ^2^ > 2σ(*F* ^2^)] = 0.054
*wR*(*F* ^2^) = 0.181
*S* = 1.13
1030 reflections
103 parameters
H atoms treated by a mixture of independent and constrained refinement
Δρ_max_ = 0.29 e Å^−3^
Δρ_min_ = −0.31 e Å^−3^

### 

Data collection: *APEX2* (Bruker, 2009 ▶); cell refinement: *SAINT* (Bruker, 2009 ▶); data reduction: *SAINT*; program(s) used to solve structure: *SHELXTL* (Sheldrick, 2008 ▶); program(s) used to refine structure: *SHELXTL*; molecular graphics: *SHELXTL*; software used to prepare material for publication: *SHELXTL* and *PLATON* (Spek, 2009 ▶).

## Supplementary Material

Crystal structure: contains datablock(s) global, I. DOI: 10.1107/S1600536814020285/sj5423sup1.cif

Structure factors: contains datablock(s) I. DOI: 10.1107/S1600536814020285/sj5423Isup2.hkl

Supporting information file. DOI: 10.1107/S1600536814020285/sj5423Isup3.cml

**Fig. 1.**
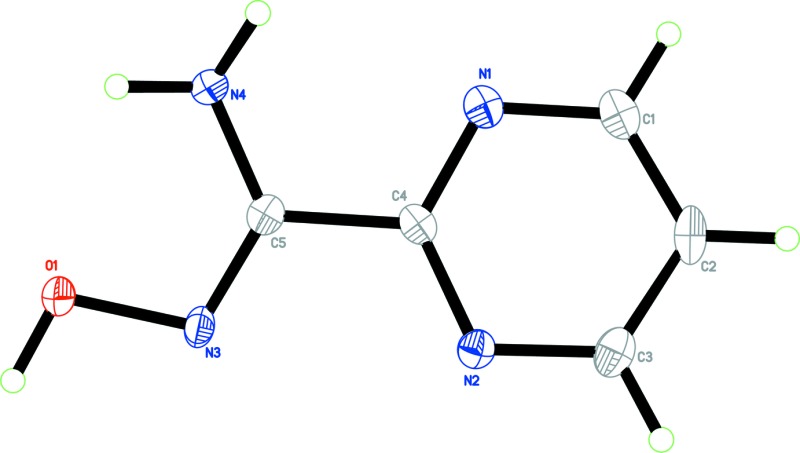
The molecular structure of the title compound with atom labels. Displacement ellipsoids are shown at the 50% probability level.

**Fig. 2.**
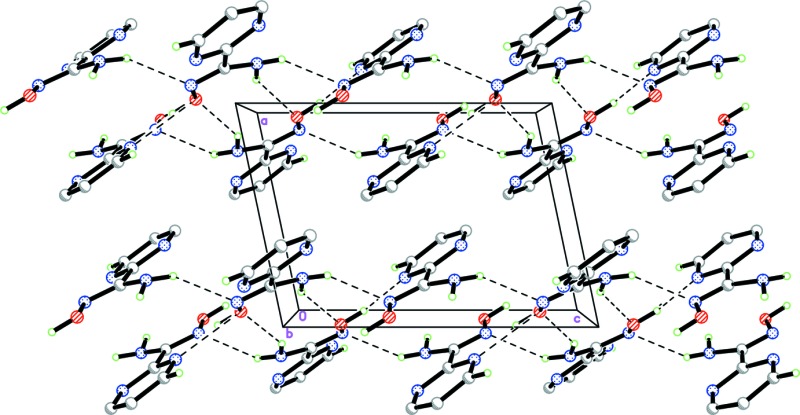
The crystal packing of the title compound viewed along the b axis die=rection. H atoms not involved in intermolecular interactions (dashed lines) have been omitted for clarity.

. DOI: 10.1107/S1600536814020285/sj5423fig1.tif
The mol­ecular structure of the title compound with atom labels. Displacement ellipsoids are shown at the 50% probability level.

b . DOI: 10.1107/S1600536814020285/sj5423fig2.tif
The crystal packing of the title compound viewed along the *b* axis die=rection. H atoms not involved in inter­molecular inter­actions (dashed lines) have been omitted for clarity.

CCDC reference: 1018015

Additional supporting information:  crystallographic information; 3D view; checkCIF report

## Figures and Tables

**Table 1:** Hydrogen-bond geometry (Å, °)

*D*—H⋯*A*	*D*—H	H⋯*A*	*D*⋯*A*	*D*—H⋯*A*
N4—H2*N*4⋯O1^i^	0.89 (3)	2.27 (3)	2.996 (3)	139 (3)
N4—H1*N*4⋯N3^ii^	0.92 (3)	2.30 (3)	3.106 (3)	146 (3)
O1—H1*O*1⋯N2^iii^	0.95 (4)	1.85 (4)	2.783 (3)	167 (3)
C3—H3*A*⋯O1^iv^	0.95	2.51	3.305 (4)	141

Symmetry codes: (i) 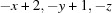; (ii) 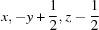; (iii) 
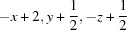
; (iv) 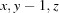.

## supplementary crystallographic information

### S1. Comment

Supramolecular architectures assembled *via* various delicate noncovalent interactions such as hydrogen bonds, π—π stacking and electrostatic interactions, have attracted intense interest in recent years because of their fascinating structural diversity and potential applications for functional materials (Desiraju, 2007). In particular, the application of intermolecular hydrogen bonding is a well known and efficient tool in the field of organic crystal design owing to their strength and directional properties (Aakeroy & Seddon, 1993). Substituted *N*'-hydroxybenzamidines are important intermediates obtained during the synthesis of pharmaceuticaly important 1,2,4-oxadiazole derivatives (Kundu *et al.*, 2012). 1,2,4-oxadiazole derivatives are well known for their biological activities such as for anti-HIV (Sakamoto *et al.*, 2007) and anti-microbial applications (Tyrkov & Sukhenko, 2004). Herein, we report the crystal structure determination of the title compound, (I).

The asymmetric unit of the title compound is shown in Fig. 1. The essentially planar pyrimidine ring [N1/N2/C1–C4, maximum deviation of 0.009 (2) Å at atom C4] forms a dihedral angle of 11.04 (15)° with the hydroxyacetimidamide (N4/C5/N3/O1). The compound adopts an *E* configuration across the C5═N3 double bond, as the OH group and benzene ring are on opposite sides of the double bond while the hydrogen atom of the hydroxy group is directed away from the NH_2_ group. The bond lengths and angles are within normal ranges.

In the crystal packing, molecules are linked by a pair of N4—H2N4···O1^i^ hydrogen bonds (symmetry code in Table 1) into an inversion dimer, forming an R_2_^2^(10) ring motif. These molecules are self-assembled *via* N4—H1N4···N3^ii^ hydrogen bonds (graph-set notation C(4); symmetry code as in Table 1), which interconnect the dimers resulting in a sheet parallel to the *ac* plane as shown in Fig 2. Furthermore, the crystal structure is stabilized by O1–H1O1···N2^iii^ and weak C3—H3A···O1^iv^ hydrogen bonds (symmetry code as in Table 1) and π–π stacking interactions between the pyrimidine (N1/N2/C1-C4) rings [centroid-centroid distance = 3.622 (1) Å; (symmetry code: 1-*x*, - *y*, -*z*)].

### S2. Experimental

A hot methanol solution (20 ml) of *N*'-hydroxypyrimidine- 2-carboximidamide (69 mg, Aldrich) was warmed over a magnetic stirrer hotplate for a few minutes. The resulting solution was allowed to cool slowly at room temperature. Single crystals of the title compound (I) appeared from the mother liquor after a few days.

### S3. Refinement

O- and N-bound H atoms were located in a difference Fourier map and refined freely [O–H = 0.94 (4) Å and N–H = 0.89 (3) Å and 0.92 (3) Å]. The remaining hydrogen atoms were positioned geometrically [C–H= 0.95 Å] and were refined using a riding model, with *U*_iso_(H)=1.2 *U*_eq_(C).

### Crystal data

**Table:**

C_5_H_6_N_4_O	*F*(000) = 288
*M**_r_* = 138.14	*D*_x_ = 1.545 Mg m^−^^3^
Monoclinic, *P*2_1_/*c*	Mo *K*α radiation, λ = 0.71073 Å
Hall symbol: -P 2ybc	Cell parameters from 1905 reflections
*a* = 7.4066 (7) Å	θ = 2.8–29.9°
*b* = 8.0165 (8) Å	µ = 0.12 mm^−^^1^
*c* = 10.2200 (9) Å	*T* = 100 K
β = 101.888 (6)°	Plate, colourless
*V* = 593.8 (1) Å^3^	0.62 × 0.17 × 0.08 mm
*Z* = 4	

### Data collection

**Table:**

Bruker SMART APEXII CCD area-detector diffractometer	1030 independent reflections
Radiation source: fine-focus sealed tube	831 reflections with *I* > 2σ(*I*)
Graphite monochromator	*R*_int_ = 0.047
φ and ω scans	θ_max_ = 25.0°, θ_min_ = 2.8°
Absorption correction: multi-scan (*SADABS*; Bruker, 2009)	*h* = −8→8
*T*_min_ = 0.931, *T*_max_ = 0.990	*k* = −9→8
4073 measured reflections	*l* = −12→12

### Refinement

**Table:**

Refinement on *F*^2^	Primary atom site location: structure-invariant direct methods
Least-squares matrix: full	Secondary atom site location: difference Fourier map
*R*[*F*^2^ > 2σ(*F*^2^)] = 0.054	Hydrogen site location: inferred from neighbouring sites
*wR*(*F*^2^) = 0.181	H atoms treated by a mixture of independent and constrained refinement
*S* = 1.13	*w* = 1/[σ^2^(*F*_o_^2^) + (0.093*P*)^2^ + 0.7207*P*] where *P* = (*F*_o_^2^ + 2*F*_c_^2^)/3
1030 reflections	(Δ/σ)_max_ < 0.001
103 parameters	Δρ_max_ = 0.29 e Å^−^^3^
0 restraints	Δρ_min_ = −0.31 e Å^−^^3^

### Special details

**Table:**

Experimental. The crystal was placed in the cold stream of an Oxford Cryosystems Cobra open-flow nitrogen cryostat operating at 100.0 (1) K.
Geometry. All e.s.d.'s (except the e.s.d. in the dihedral angle between two l.s. planes) are estimated using the full covariance matrix. The cell e.s.d.'s are taken into account individually in the estimation of e.s.d.'s in distances, angles and torsion angles; correlations between e.s.d.'s in cell parameters are only used when they are defined by crystal symmetry. An approximate (isotropic) treatment of cell e.s.d.'s is used for estimating e.s.d.'s involving l.s. planes.
Refinement. Refinement of *F*^2^ against ALL reflections. The weighted *R*-factor *wR* and goodness of fit *S* are based on *F*^2^, conventional *R*-factors *R* are based on *F*, with *F* set to zero for negative *F*^2^. The threshold expression of *F*^2^ > σ(*F*^2^) is used only for calculating *R*-factors(gt) *etc*. and is not relevant to the choice of reflections for refinement. *R*-factors based on *F*^2^ are statistically about twice as large as those based on *F*, and *R*- factors based on ALL data will be even larger.

### Fractional atomic coordinates and isotropic or equivalent isotropic displacement parameters (Å^2^)

**Table:**

	*x*	*y*	*z*	*U*_iso_*/*U*_eq_	
O1	0.9616 (3)	0.4128 (2)	0.15856 (18)	0.0171 (6)	
N1	0.6515 (3)	0.0148 (3)	−0.1252 (2)	0.0159 (6)	
N2	0.8106 (3)	−0.0836 (3)	0.0883 (2)	0.0140 (6)	
N3	0.9064 (3)	0.2423 (3)	0.1497 (2)	0.0150 (6)	
N4	0.8007 (3)	0.3212 (3)	−0.0758 (2)	0.0155 (6)	
C1	0.5875 (4)	−0.1406 (4)	−0.1538 (3)	0.0183 (7)	
H1A	0.5107	−0.1612	−0.2389	0.022*	
C2	0.6283 (4)	−0.2713 (4)	−0.0652 (3)	0.0198 (7)	
H2A	0.5801	−0.3800	−0.0867	0.024*	
C3	0.7425 (4)	−0.2368 (4)	0.0560 (3)	0.0183 (7)	
H3A	0.7743	−0.3244	0.1191	0.022*	
C4	0.7592 (3)	0.0362 (3)	−0.0043 (2)	0.0127 (6)	
C5	0.8291 (3)	0.2095 (3)	0.0268 (2)	0.0126 (7)	
H2N4	0.869 (4)	0.413 (4)	−0.057 (3)	0.012 (7)*	
H1N4	0.784 (4)	0.281 (4)	−0.162 (3)	0.025 (8)*	
H1O1	1.029 (5)	0.429 (4)	0.247 (4)	0.022 (8)*	

### Atomic displacement parameters (Å^2^)

**Table:**

	*U*^11^	*U*^22^	*U*^33^	*U*^12^	*U*^13^	*U*^23^
O1	0.0221 (11)	0.0123 (11)	0.0157 (11)	−0.0038 (8)	0.0012 (9)	−0.0005 (7)
N1	0.0142 (12)	0.0173 (13)	0.0169 (12)	−0.0003 (10)	0.0048 (10)	−0.0023 (9)
N2	0.0130 (12)	0.0135 (12)	0.0156 (12)	0.0008 (9)	0.0033 (10)	0.0000 (9)
N3	0.0164 (12)	0.0098 (12)	0.0185 (12)	−0.0026 (10)	0.0027 (10)	−0.0001 (9)
N4	0.0196 (13)	0.0134 (13)	0.0130 (12)	−0.0015 (11)	0.0023 (10)	0.0016 (9)
C1	0.0139 (14)	0.0209 (15)	0.0212 (14)	−0.0018 (12)	0.0058 (12)	−0.0060 (12)
C2	0.0166 (15)	0.0129 (14)	0.0319 (16)	−0.0030 (11)	0.0096 (13)	−0.0071 (12)
C3	0.0177 (15)	0.0147 (14)	0.0246 (15)	0.0015 (12)	0.0096 (12)	0.0004 (11)
C4	0.0092 (13)	0.0160 (15)	0.0138 (13)	0.0006 (11)	0.0043 (11)	−0.0032 (10)
C5	0.0099 (13)	0.0137 (14)	0.0160 (13)	0.0011 (11)	0.0065 (11)	0.0002 (10)

### Geometric parameters (Å, º)

**Table:**

O1—N3	1.424 (3)	N4—H2N4	0.89 (3)
O1—H1O1	0.94 (4)	N4—H1N4	0.92 (3)
N1—C4	1.336 (3)	C1—C2	1.378 (4)
N1—C1	1.343 (4)	C1—H1A	0.9500
N2—C3	1.343 (4)	C2—C3	1.376 (4)
N2—C4	1.347 (3)	C2—H2A	0.9500
N3—C5	1.295 (3)	C3—H3A	0.9500
N4—C5	1.362 (3)	C4—C5	1.494 (4)
			
N3—O1—H1O1	106.1 (18)	C3—C2—H2A	121.6
C4—N1—C1	116.0 (2)	C1—C2—H2A	121.6
C3—N2—C4	116.2 (2)	N2—C3—C2	122.4 (3)
C5—N3—O1	108.7 (2)	N2—C3—H3A	118.8
C5—N4—H2N4	112.9 (18)	C2—C3—H3A	118.8
C5—N4—H1N4	118 (2)	N1—C4—N2	125.9 (2)
H2N4—N4—H1N4	117 (3)	N1—C4—C5	115.6 (2)
N1—C1—C2	122.8 (2)	N2—C4—C5	118.5 (2)
N1—C1—H1A	118.6	N3—C5—N4	125.5 (2)
C2—C1—H1A	118.6	N3—C5—C4	117.4 (2)
C3—C2—C1	116.7 (3)	N4—C5—C4	117.1 (2)
			
C4—N1—C1—C2	0.2 (4)	C3—N2—C4—C5	179.3 (2)
N1—C1—C2—C3	−1.1 (4)	O1—N3—C5—N4	−1.7 (3)
C4—N2—C3—C2	0.8 (4)	O1—N3—C5—C4	−179.51 (19)
C1—C2—C3—N2	0.5 (4)	N1—C4—C5—N3	168.3 (2)
C1—N1—C4—N2	1.3 (4)	N2—C4—C5—N3	−12.7 (3)
C1—N1—C4—C5	−179.8 (2)	N1—C4—C5—N4	−9.7 (3)
C3—N2—C4—N1	−1.8 (4)	N2—C4—C5—N4	169.3 (2)

### Hydrogen-bond geometry (Å, º)

**Table:**

*D*—H···*A*	*D*—H	H···*A*	*D*···*A*	*D*—H···*A*
N4—H2*N*4···O1^i^	0.89 (3)	2.27 (3)	2.996 (3)	139 (3)
N4—H1*N*4···N3^ii^	0.92 (3)	2.30 (3)	3.106 (3)	146 (3)
O1—H1*O*1···N2^iii^	0.95 (4)	1.85 (4)	2.783 (3)	167 (3)
C3—H3*A*···O1^iv^	0.95	2.51	3.305 (4)	141

Symmetry codes: (i) −*x*+2, −*y*+1, −*z*; (ii) *x*, −*y*+1/2, *z*−1/2; (iii) −*x*+2, *y*+1/2, −*z*+1/2; (iv) *x*, *y*−1, *z*.

## References

[bb1] Aakeroy, C. B. & Seddon, K. R. (1993). *Chem. Soc. Rev.* **22**, 397–407.

[bb2] Bruker (2009). *SADABS*, *APEX2* and *SAINT* Bruker AXS Inc., Madison, Wisconsin, USA.

[bb3] Desiraju, G. R. (2007). *Angew. Chem. Int. Ed.* **46**, 8342–8356. 10.1002/anie.200700534 17902079

[bb4] Kundu, M., Singh, B., Ghosh, T., Maiti, B. C. & Maity, T. K. (2012). *Indian J. Chem. Sect. B*, **51**, 493–497.

[bb5] Sakamoto, T., Cullen, M. D., Hartman, T. L., Watson, K. M., Buckheit, R. W., Pannecouque, C., DeClercq, E. & Cushman, M. (2007). *J. Med. Chem.* **50**, 3314–3319. 10.1021/jm070236e PMC2531242 17579385

[bb6] Sheldrick, G. M. (2008). *Acta Cryst.* A**64**, 112–122. 10.1107/S0108767307043930 18156677

[bb7] Spek, A. L. (2009). *Acta Cryst.* D**65**, 148–155. 10.1107/S090744490804362X PMC2631630 19171970

[bb8] Tyrkov, A. G. & Sukhenko, L. T. (2004). *Pharm. Chem. J.* **38**, 30–38.

