# Supplementary figures and images for: Crystal structure of N′-hy­droxy­pyrimidine-2-carboximidamide
Source: Acta Crystallogr Sect E Struct Rep Online. 2014 Sep 13;70(Pt 10):o1107–8. doi: 10.1107/S1600536814020285 (PMC4257198; doi:10.1107/S1600536814020285)

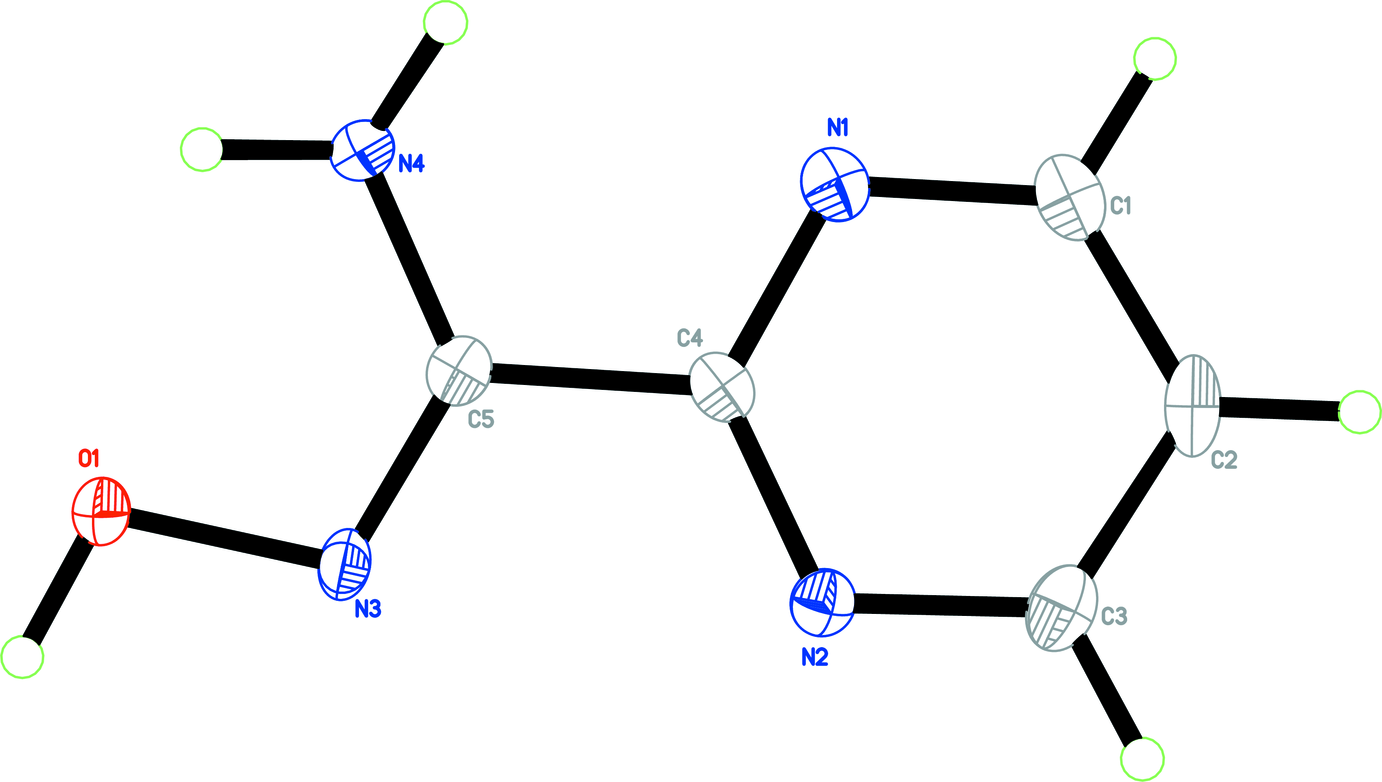

Supplement: Supplementary file 4 [file e-70-o1107-fig1.tif]

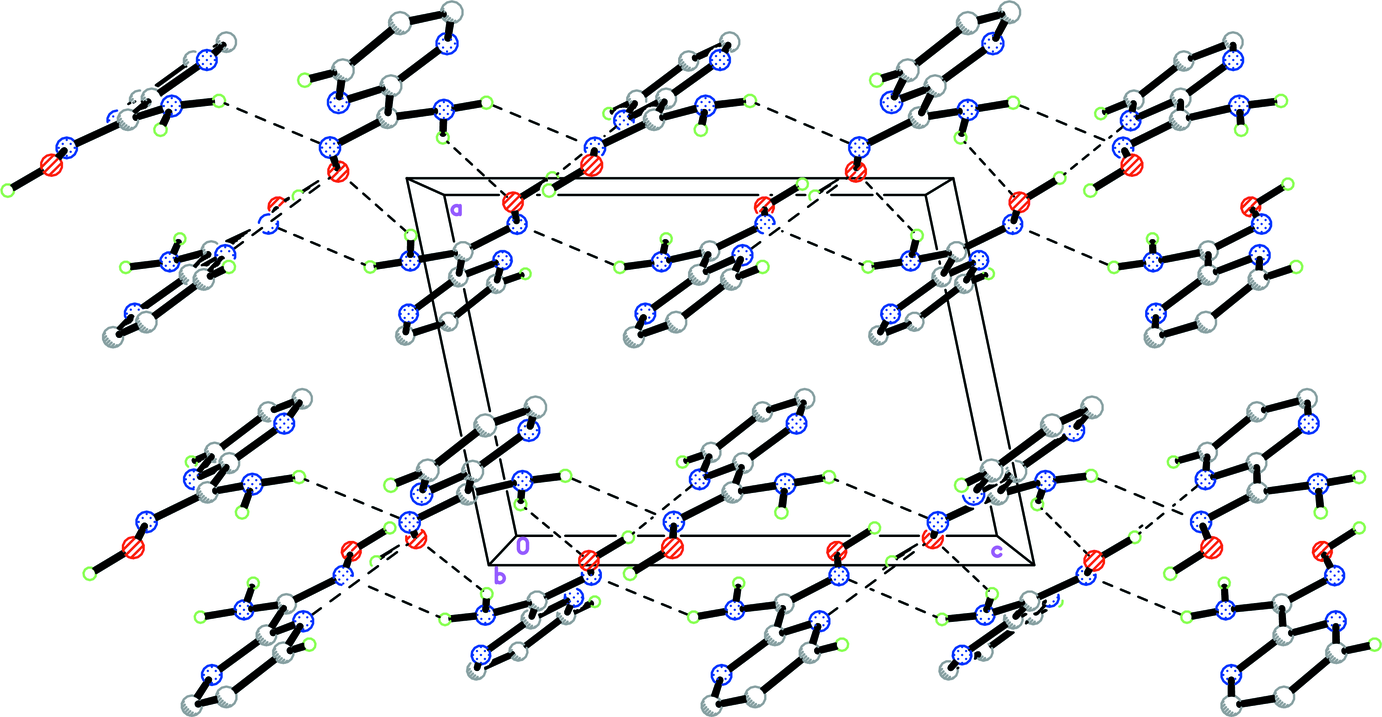

Supplement: Supplementary file 5 [file e-70-o1107-fig2.tif]
